# Epigallocatechin-3-gallate (EGCG) up-regulates miR-15b expression thus attenuating store operated calcium entry (SOCE) into murine CD4^+^ T cells and human leukaemic T cell lymphoblasts

**DOI:** 10.18632/oncotarget.20032

**Published:** 2017-08-08

**Authors:** Shaqiu Zhang, Tamer al-Maghout, Rosi Bissinger, Ni Zeng, Lisann Pelzl, Madhuri S. Salker, Anchun Cheng, Yogesh Singh, Florian Lang

**Affiliations:** ^1^ Institute of Preventive Veterinary Medicine, Sichuan Agricultural University, Wenjiang, Chengdu, Sichuan, P.R. China; ^2^ Department of Internal Medicine III, Tübingen University, Gmelinstraβe, Tübingen, Germany; ^3^ Department of Cleft Lip and Palate Surgery, State Key Laboratory of Oral Diseases, National Clinical Research Center for Oral Diseases, West China Hospital of Stomatology, Sichuan University, Chengdu, P.R. China; ^4^ Institute of Women’s Health, Tübingen University, Calwerstraβe, Tübingen, Germany; ^5^ Institute of Medical Genetics and Applied Genomics, Tübingen University, Calwerstraβe, Tübingen, Germany; ^6^ Institute of Physiology I, Tübingen University, Gmelinstraβe, Tübingen, Germany

**Keywords:** Murine CD4^+^ T cells, human leukaemic T cell lymphoblasts, EGCG, SOCE, miR-15b, Immunology and Microbiology Section, Immune response, Immunity

## Abstract

CD4^+^ T cells are key elements in immune responses and inflammation. Activation of T cell receptors in CD4^+^ T cells triggers cytosolic Ca^2+^ release with subsequent store operated Ca^2+^ entry (SOCE), which is accomplished by the pore forming Ca^2+^ release activated Ca^2+^ (CRAC) channel Orai1 and its regulator stromal cell-interaction molecule 2 (STIM2). Green tea polyphenol epigallocatechin-3-gallate (EGCG) acts as a potent anti-inflammatory and anti-oxidant agent for various types of cells including immune cells. However, how post-transcriptional gene regulators such as miRNAs are involved in the regulation of Ca^2+^ influx into murine CD4^+^ T cells and human Jurkat T cells through EGCG is not defined. EGCG treatment of murine CD4^+^ T cells significantly down-regulated the expression of STIM2 and Orai1 both at mRNA and protein levels. Furthermore, EGCG significantly decreased SOCE in both murine and human T cells. EGCG treatment increased miRNA-15b (miR-15b) abundance in both murine and human T cells. Bioinformatics analysis reveals that miR-15b, which has a STIM2 binding site, is involved in the down-regulation of SOCE. Overexpression of miR-15b significantly decreased the mRNA and protein expression of STIM2 and Orai1 in murine T cells. Treatment of Jurkat T cells with 10 μM EGCG further decreased mTOR and PTEN protein levels. EGCG decreased mitochondrial membrane potential (MMP) in both human and murine T cells. In conclusion, the observations suggest that EGCG inhibits the Ca^2+^ entry into murine and human T cells, an effect accomplished at least in part by up-regulation of miR-15b.

## INTRODUCTION

During immune responses, antigen-specific T cell activation with induction of gene expression, proliferation, cell motility and cytokine expression requires a sudden surge in intracellular Ca^2+^ levels [1–3]. In the resting state of T cells, Ca^2+^ is stored in the endoplasmic reticulum (ER), where it is sensed by stromal cell-interaction molecules (STIM) 1 and 2. Activation of the T cell receptor results in the production of inositol trisphosphate (IP_3_), which binds to IP_3_ receptors on the ER thus triggering the release of Ca^2+^ into the cytosol [1]. The emptying of the intracellular Ca^2+^ stores is followed by activation of store-operated Ca^2+^ entry (SOCE) [1–5] accomplished by activation of calcium release-activated calcium (CRAC) channel protein 1 (encoded by Orai1 gene) by the ER Ca^2+^ sensors STIM1 and 2. Ca^2+^ influx through Orai1 in T cells depends on a negative membrane potential that provides the electrical driving force for Ca^2+^ entry [1, 4, 6–8].

Inhibitors of immune responses and inflammation include the green tea polyphenol epigallocatechin-3-gallate (EGCG) [9, 10]. Green tea consumption is effective against various clinical disorders such as autoimmunity, cardiovascular disease, obesity and neurodegenerative disease [9–17]. In many cell types EGCG impacts on Ca^2+^ regulation, intracellular pH regulation, cell proliferation, cell death, and cytokine secretion [9–17]. Excessive rise in ([Ca^2+^]i produces adverse effects and results in cell death [18]. Hence, it is indispensable for cells to delicately buffer ([Ca^2+^]i and to precisely regulate the entry of Ca^2+^ [19]. In AßPP/PS1 (presenilin 1) double mutant transgenic mouse model of Alzheimer's disease, EGCG restored mitochondrial respiratory rates, mitochondrial membrane potential (MMP), reactive oxygen species (ROS) and ATP levels in several brain regions including hippocampus, cortex, and striatum [20]. EGCG protects cells not only by counteracting oxidative stress but also by modulating signalling pathways, cell survival and cell death genes [18]. EGCG increases expression levels of PKC, thus boosting PKC signalling with activation of Bcl-2, extracellular signal related kinases-ERK 1, ERK 2 and reduction in the levels of proapoptotic caspase 6, bax, bad, TRAIL and Fas ligand [18, 21, 22]. EGCG activates the metabolic regulator adenosine 5’-monophosphate-activated protein kinase (AMPK) in mesangial cells and inhibits the immune-stimulated phosphoinositide 3-kinase (PI3K)/Akt/mammalian target of rapamycin (mTOR) pathway [22, 23] through its inhibitor phosphatase and tensin homolog deleted on chromosome 10 (PTEN) [24].

Recent studies have suggested that microRNAs (miRNAs) not only play a vital role in maintaining cellular functions but are also involved in cancer development [25, 26]. The miRNAs regulate eukaryotic gene expression by cytoplasmic control of mRNA translation and degradation [27]. As shown in MCF-7 or hepatocellular carcinoma HepG3 cancer cells treatment with EGCG may up-regulate or down-regulate several miRNAs [10, 15, 25]. For instance, EGCG treatment of MCF-7 and hepatocellular carcinoma HepG3 cancer cells up-regulates miR-16, which in turn contributes to triggering of tumor cell death [10, 15, 18, 25].

The present study explored whether EGCG regulates miRNAs expression and SOCE in murine CD4^+^ T cells as well as in human leukaemic T cell lymphoblasts (Jurkat T cells). Our results suggest that in murine CD4^+^ T cells green tea polyphenol EGCG down-regulates the expression of STIM2 and Orai1 thus reducing SOCE and in a similar fashion Jurkat T cells also down-regulates the SOCE. It is further shown that EGCG up-regulates expression of miR-15b, which in turn down-regulates STIM2 and Orai1 at mRNA transcript and protein levels thus blunting SOCE in murine CD4^+^ T cells. Similarly, miR-15b was also found to be up-regulated in Jurkat T cells after 10 μM EGCG treatment. Treatment of Jurkat T cells with 10 μM EGCG decreased mTOR and PTEN protein levels. Additionally, EGCG treatment decreased mitochondrial membrane potential (MMP) in Jurkat T cells and murine CD4^+^ T cells. Hence, the present observations uncover a novel action of EGCG, i.e. the up-regulation of miR-15b and down-regulation of mitochondrial membrane potential and store operated Ca^2+^ influx into murine CD4^+^ T cells and Jurkat T cells.

## RESULTS

### Effect of EGCG on apoptosis and cell proliferation in activated murine CD4^+^ T cells

**Figure 1:**
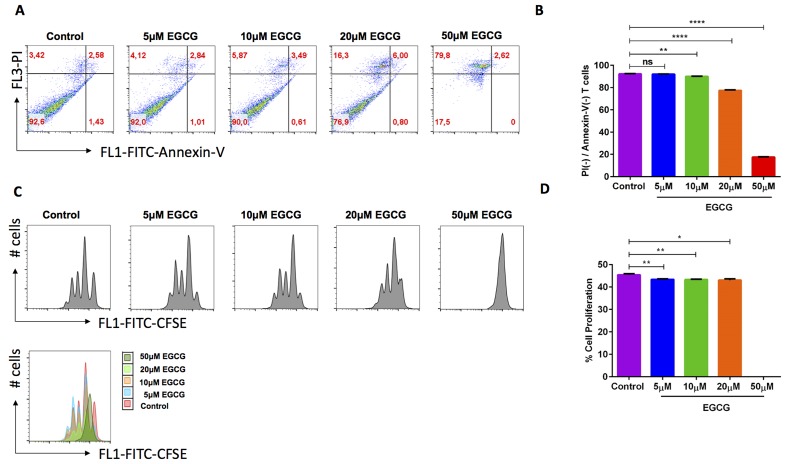
EGCG significantly enhanced cell death and decreased cell proliferation of murine CD4 **+** T cells. Murine CD4^+^ naïve T cells were isolated from C57BL/6 mice (spleen and lymph nodes). Apoptosis as well as cell proliferation were measured by flow cytometry. **A.** Murine CD4^+^ naïve T cells were activated in the presence of anti-CD3/anti-CD28 and cultured in the presence of (5-50 μM) EGCG for 3 days. Apoptosis was determined using Annexin-V and Propidium Iodide staining by flow cytometry. **B.** No significant difference in apoptosis was observed between control (violet bar) and 5 μM EGCG (blue bar) treated murine CD4^+^ T cells. However, treatment with 10 μM (green bar), 20 μM (orange bar), and 50 μM (red bar) EGCG had also significantly increased cell death. Data are representative for 4 independent experiments. Arithmetic means ± SEM (*n* = 4) of live cells after treatment with different concentrations of EGCG (5-50 μM). **(*p* < 0.01), ****(*p* < 0.0001) indicates statistically significant difference when compared with control. **C.** Murine CD4^+^ naïve T cells were stained with CFSE dye before activation with anti-CD3/anti-CD28 and cultured in the presence of (5-50 μM) EGCG for 3 days. Cell proliferation was measured by flow cytometry. Data shown here are representative for 4 independent experiments. X-axis represents the CFSE dye whereas y-axis represents cell numbers (# no. of cells). Overlays plot of cell proliferation with different concentrations of EGCG. X-axis represents the CFSE dye whereas y-axis represents cell numbers (# no. of cells). **D.** Arithmetic means ± SEM (*n* = 4) of second peak of proliferation (first peak non-proliferated cells). Statistically significant difference in cell proliferation was observed between control and 10 μM EGCG treated murine CD4^+^ T cells. *(*p* < 0.05), **(*p* < 0.01), indicates statistically significant difference when compared with control.

Previous studies suggested that EGCG influences various fundamental cell pathways such as apoptosis, cell proliferation of various cancer cell lines and primary cells including T cells [13, 15, 16, 18, 25, 28]. We used Annexin-V and Propidium Iodide (PI) for measuring apoptosis with increasing concentrations ranging from 5 μM - 50 μM. We found that 10 μM EGCG concentration slightly enhanced early apoptosis (Figure 1A & 1B). Further, we measured cell proliferation using the CFSE dye. As a result, cell proliferation was significantly decreased in the presence of 5 - 20 μM EGCG compared with control. At 50 μM EGCG cells almost stopped proliferating (Figure 1C & 1D). We decided to use mainly 10 μM EGCG for further experiments.

### EGCG down-regulates SOCE in activated murine CD4^+^ T cells

**Figure 2:**
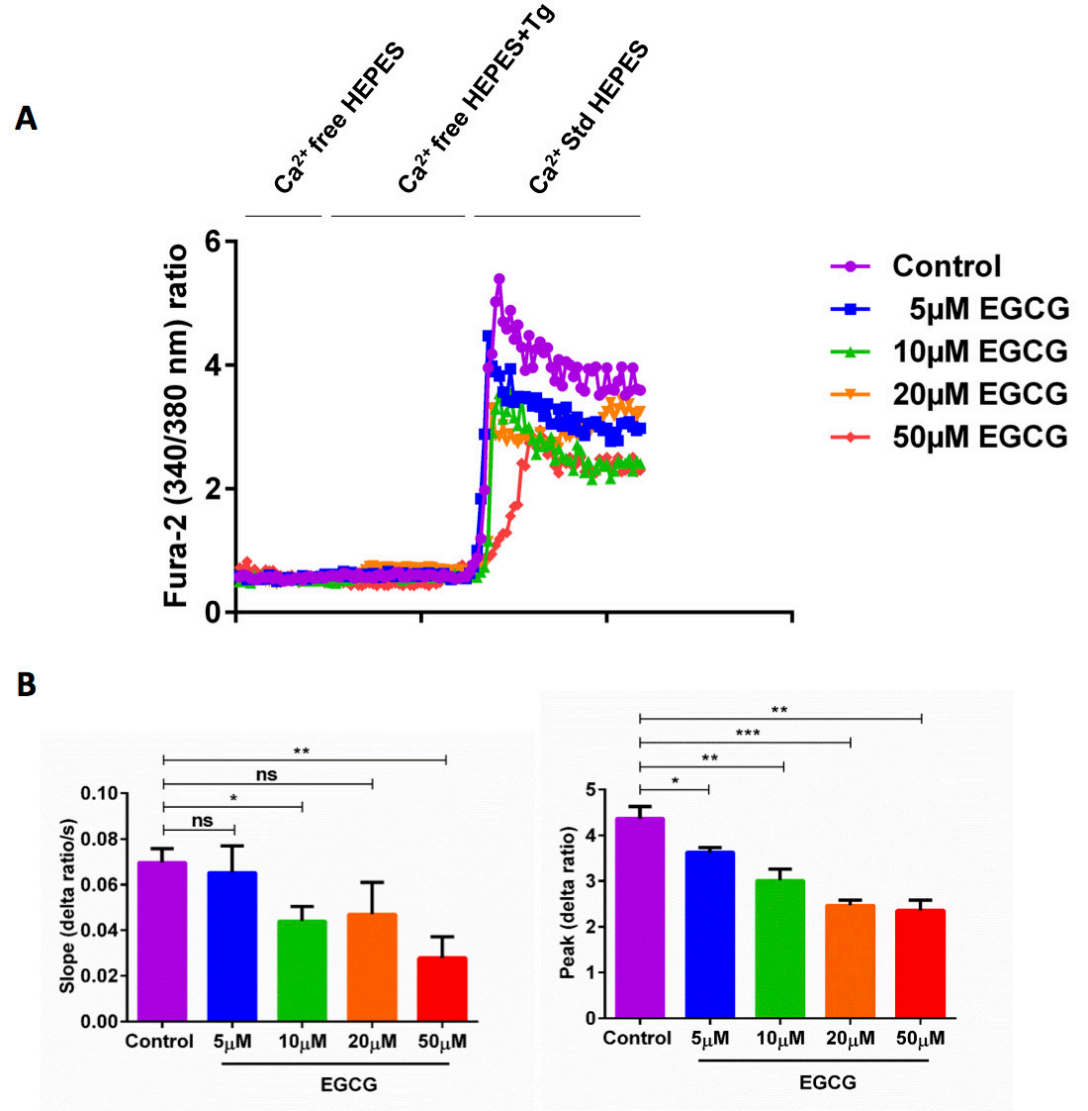
EGCG treatment significantly decreased SOCE in activated murine CD4 **^+^** T cells. **A.** Representative tracings showing the 340/380 nm fluorescence ratio reflecting cytosolic Ca^2+^ activity in Fura-2, AM loaded activated (plate bound anti-CD3 and anti-CD28) murine CD4^+^ T cells incubated for 72 hours without and with different concentration of (5-50 μM) EGCG followed by subsequent exposure to Ca^2+^-free HEPES, additional exposure to sarcoendoplasmatic Ca^2+^ ATPase (SERCA) inhibitor thapsigargin (Tg, 1 μM) and re-addition of extracellular Ca^2+^ (Ca^2+^ Std HEPES). **B.** Arithmetic means ± SEM (*n* = 4) of the slope (left) and peak (right) of the fluorescence ratio change following re-addition of extracellular Ca^2+^ in murine CD4^+^ T cells incubated for 72 hours without (violet bars) and with 5 μM (blue bars), 10 μM (green bars), 20 μM (orange bars), and 50 μM (red bars) EGCG. *(*p* < 0.05), **(*p* < 0.01), ***(*p* < 0.001) indicates statistically significant difference.

Orai1 channels, stimulated by STIM2, accomplish store operated Ca^2+^ entry (SOCE) into CD4^+^ T cells and are thus decisive for T cell activation [1]. To quantify the intracellular Ca^2+^ activity ([Ca^2+^]i and SOCE from control and EGCG treated murine CD4^+^ T cells, Fura-2 fluorescence was determined. CD4^+^ T cells were activated for 3 days in the presence of plate-bound anti-CD3 and anti-CD28 (1:2 ratio) and in the presence or absence of EGCG (5 - 50 μM). The activated cells were loaded with Fura-2 for 30 minutes in standard HEPES and washed once with standard HEPES. [Ca^2+^]i was measured first in standard HEPES, which was subsequently replaced by Ca^2+^-free HEPES. In a next step the intracellular Ca^2+^ stores were depleted by addition of sarco-/endoplasmic reticulum Ca^2+^ ATPase (SERCA) inhibitor thapsigargin (1μM) in the nominal absence of extracellular Ca^2+^. The subsequent re-addition of extracellular Ca^2+^ was followed by a sharp increase of [Ca^2+^]i. Both, slope and peak of the [Ca^2+^]i increase were significantly lower in 10 μM EGCG treated cells than in control murine CD4^+^ T cells (Figure 2). Increasing the EGCG concentrations (20 μM and 50 μM) did not further decrease SOCE, when compared with 10 μM EGCG. Whereas at the lower concentrations (5 μM) of EGCG, the slope of the [Ca^2+^]i increase was almost the same in 5 μM EGCG treated cells and in control cells, the peak of the [Ca^2+^]i increase was significantly lower in 5 μM EGCG treated cells than in control cells (Figure 2).

### EGCG down-regulates the expression of STIM2 and Orai1 in activated murine CD4^+^ T cells

**Figure 3:**
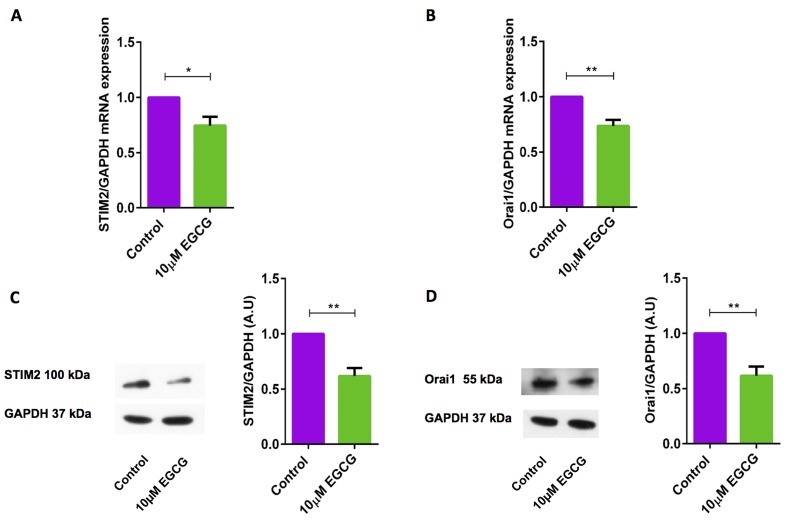
EGCG significantly decreased STIM2 and Orai1 transcript levels and protein abundance in murine CD4 **^+^** T cells. **A.**, **B.** Arithmetic means ± SEM (*n* = 3) of (A) STIM2/GAPDH and (B) Orai1/GAPDH transcript levels in murine CD4^+^ T cells following a 72 hours incubation without (violet bars) and with (green bars) 10 μM EGCG. *(*p* < 0.05), **(*p* < 0.01) indicates statistically significant difference. **C.**, **D.** Original Western blots (left panels) and arithmetic means ± SEM (*n* = 4-5, right panels) of (C) STIM2/GAPDH and (D) Orai1/GAPDH protein abundance in murine CD4^+^ T cells following a 72 hours incubation without (control, violet bars) and with (EGCG, green bars) 10 μM EGCG. **(*p* < 0.01) indicates statistically significant difference.

The previous results indicated that SOCE (both peak and slope) was significantly down-regulated after 10 μM EGCG in murine CD4^+^ T cells. Hence, utilizing qRT-PCR, we explored whether EGCG influences STIM2 and/or Orai1 transcript levels in murine CD4^+^ T cells. As illustrated in Figure 3A & 3B, treatment of CD4^+^ T cells with 10 μM EGCG for 72 hours significantly decreased STIM2 and Orai1 mRNA abundance. Western blotting was employed to test, whether the effect of EGCG on transcript levels was paralleled by similar effects on protein abundance. As shown in Figure 3C & 3D, EGCG treatment indeed significantly decreased STIM2 and Orai1 protein expression.

### EGCG treatment augments the miR-15b expression in activated murine CD4^+^ T cells

**Figure 4:**
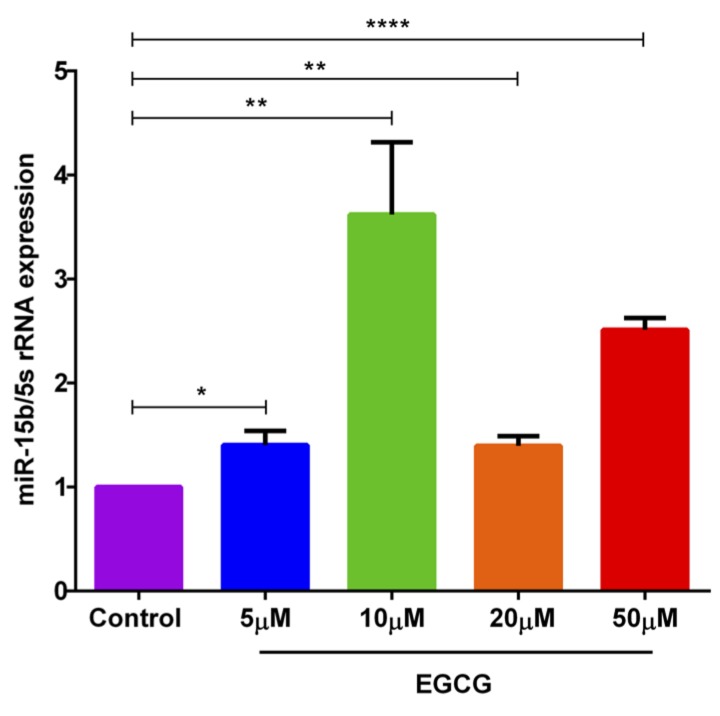
EGCG treatment significantly increased miR-15b expression in murine CD4 **^+^** T cells. Arithmetic means ± SEM (*n* = 4-8) of miR-15b over 5S rRNA transcript levels in murine CD4^+^ T cells following a 72 hours incubation without (violet bar) and with 5 μM (blue bar), 10 μM (green bar), 20 μM (orange bar), and 50 μM (red bar) EGCG. *(*p* < 0.05), **(*p* < 0.01), ****(*p* < 0.0001) indicates statistically significant difference.

We sought to determine, whether EGCG influences miR-15b abundance, we measured the miR-15b expression in murine CD4^+^ T cells after treatment with EGCG (5, 10, 20, 50 μM) by the miR-qRT-PCR method. As a result, treatment of murine CD4^+^ T cells with 10 μM EGCG was followed by a marked and highly significant increase of miR-15b abundance (Figure 4).

### miR-15b overexpression (miR-15b mimic transfection) decreases STIM2 and Orai1 both at mRNA transcript and protein levels in activated murine CD4^+^ T cells

**Figure 5:**
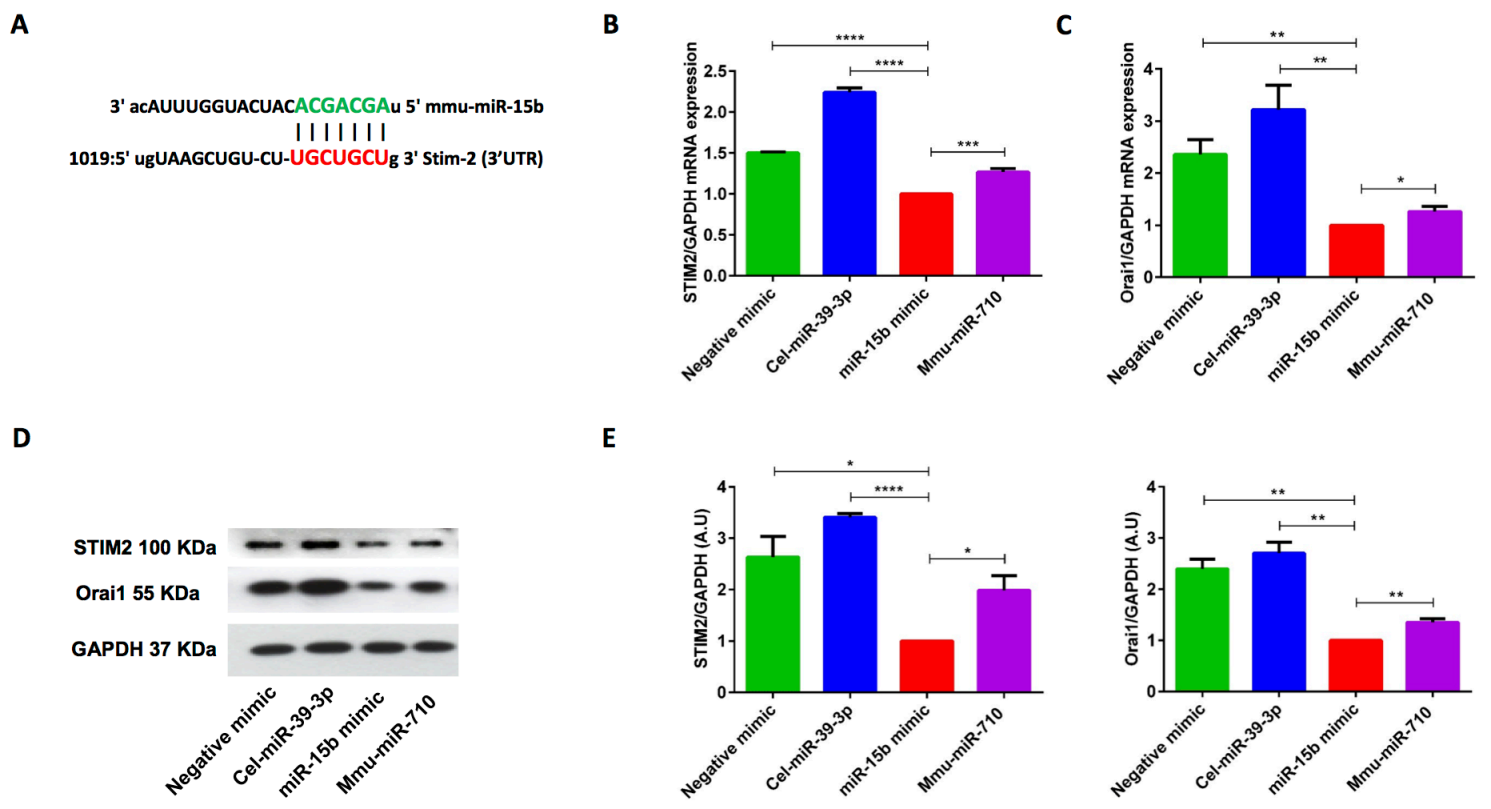
miR-15b mimic overexpression significantly decreased STIM2 and Orai1 transcript levels and protein abundance in murine CD4 **^+^** T cells. **A.** Cartoon showing the murine mmu-miR-15b (family of miR-16/miR-15a/miR-497/miR-322/miR-195) with STIM2 3’-untranslated region (3’-UTR) with seed sequence. **B.**, **C.** Arithmetic means ± SEM (*n* = 4) of (B) STIM2/GAPDH and (C) Orai1/GAPDH transcript levels in negative mimic control (green bars), cel-miR-39-3p (blue bars), miR-15b mimic (red bars) and non-specific mmu-miR-710 (violet bars) transfected murine CD4^+^ T cells. *(*p* < 0.05), **(*p* < 0.01), ***(*p* < 0.001), ****(*p* < 0.0001) indicates statistically significant difference. **D.** Original Western blots (left panels) and **E**. arithmetic means ± SEM, (*n* = 3, right panels) of STIM2/GAPDH and Orai1/GAPDH protein abundance in murine CD4^+^ T cells in negative mimic control (green bars), cel-miR-39-3p (blue bars), miR-15b mimic (red bars) and non-specific mmu-miR-710 (violet bars) transfected murine CD4^+^ T cells. *(*p* < 0.05), **(*p* < 0.01), ****(*p* < 0.0001) indicates statistically significant difference.

Bioinformatics analysis (http://www.microrna.org) suggested that murine miR-15b (mmu-miR-15b) could regulate Ca^2+^ pathways by regulating STIM1/2 proteins as miR-15b family has a strong binding site in the 3’untranslated region (3’UTR) of STIM2 (Figure 5A). To confirm whether miR-15b influenced STIM2 and/or Orai1 transcription, we transfected murine CD4^+^ T cells with negative mimic, non-specific cel-miR-39-3p control, mmu-miR-15b mimic and mmu-miR-710 (lacking the predicted binding site in STIM2 or Orai1 3’UTR region), and measured transcript levels of STIM2 and Orai1. The qRT-PCR data indeed revealed a marked and highly significant down-regulation of both STIM2 and Orai1 transcript levels following miR-15b overexpression (Figure 5B & 5C). The decreases of transcript levels were paralleled by similar alterations of protein levels. Western blot analysis further demonstrated that miR-15b overexpression was followed by down-regulation of STIM2 and Orai1 protein abundance (Figure 5D & 5E). The data supported our assertion that overexpression of miR-15b thus decreased STIM2 and Orai1 expression both at mRNA and protein levels in murine CD4^+^ T cells (Figure 5B-5E).

### miR-15b overexpression decreases SOCE in activated murine CD4^+^ T cells

**Figure 6:**
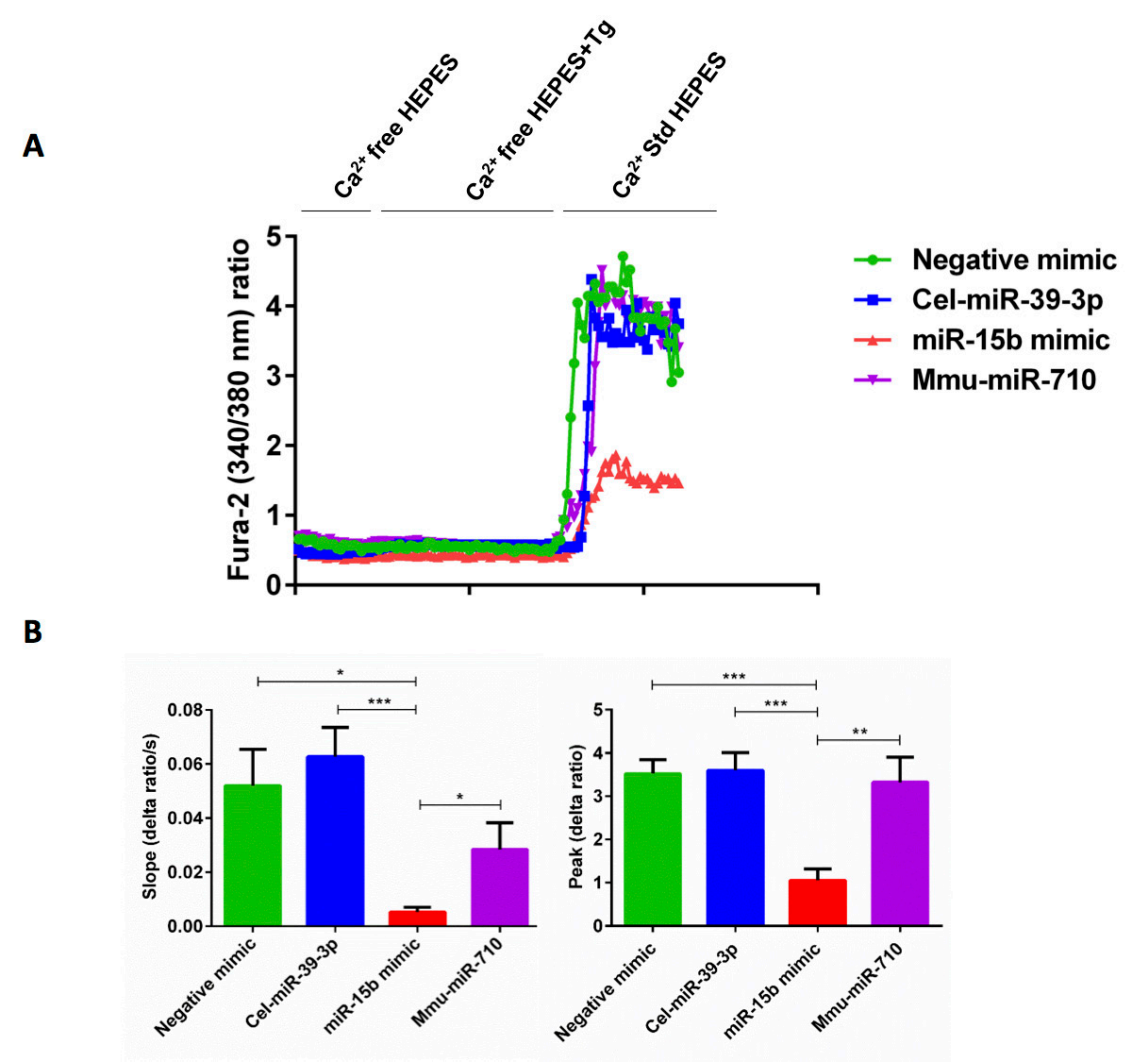
miR-15b mimic overexpression significantly decreased SOCE in activated murine CD4 **^+^** T cells. **A.** Representative tracings showing the 340/380 nm fluorescence ratio reflecting cytosolic Ca^2+^ activity in Fura-2/AM loaded negative mimic control (green curve), cel-miR-39-3p (blue curve), miR-15b mimic (red curve) and non-specific mmu-miR-710 (violet curve) transfected murine CD4^+^ T cells following exposure to Ca^2+^-free HEPES, additional exposure to Tg (1μM) and re-addition of extracellular Ca^2+^ (Ca^2+^ Std HEPES). **B.** Arithmetic means ± SEM (*n* = 3-6) of the slope (left) and peak (right) of the fluorescence ratio change following re-addition of extracellular Ca^2+^ in negative mimic control (green bars), cel-miR-39-3p (blue bars), miR-15b mimic (red bars) and non-specific mmu-miR-710 (violet bars) transfected murine CD4^+^ T cells. *(*p* < 0.05), **(*p* < 0.01), ***(*p* < 0.001) indicates statistically significant difference.

We next investigated, whether the down-regulation of STIM2 and Orai1 expression following miR-15b overexpression was paralleled by a similar decrease of SOCE. Control mimic and miR-15b mimic transfected murine CD4^+^ T cells were activated for 3 days in the presence of plate-bound anti-CD3 and anti-CD28 (1:2 ratio) and Ca^2+^ entry was measured at day 3. The activated cells were loaded with Fura-2 for 30 minutes in standard HEPES and washed once with standard HEPES. [Ca^2+^]i was measured first in standard HEPES, which was subsequently replaced by Ca^2+^-free HEPES. In a next step the intracellular Ca^2+^ stores were depleted by addition of SERCA inhibitor thapsigargin (1 μM) in the nominal absence of extracellular Ca^2+^. The subsequent re-addition of extracellular Ca^2+^ was followed by a sharp increase of [Ca^2+^]i. Both, slope and peak of the [Ca^2+^]i increase were significantly lower in miR-15b mimic transfected than in control mimic transfected murine CD4^+^ T cells (Figure 6). Non-specific cel-miR-39-3p control or mmu-miR-710 do not harbour the predicted target binding site for STIM1/2 or Orai1 and therefore, did not affect SOCE (Figure 6). Therefore, our data unequivocally suggested that miR-15b is involved in the regulation of STIM2 and Orai1 expression at the post-transcriptional level.

### EGCG augments the miR-15b expression, thus reducing SOCE, decreasing mTOR and PTEN protein levels in human Jurkat T cells

**Figure 7:**
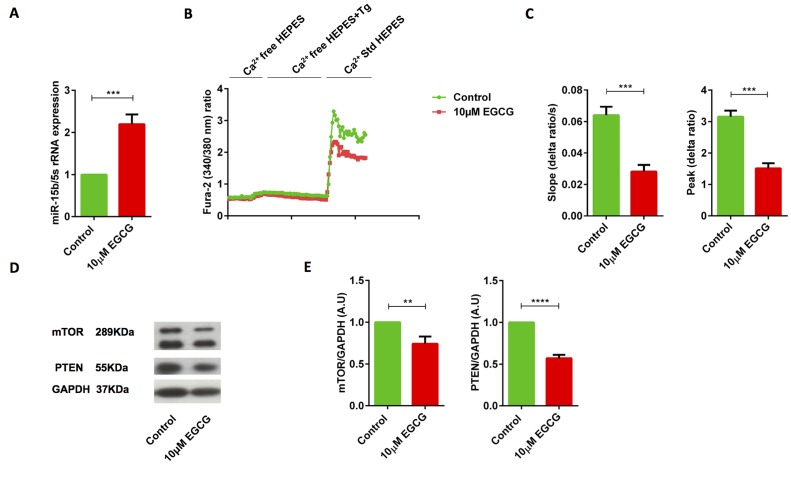
EGCG treatment significantly increased miR-15b expression, decreased SOCE, reduced PTEN and mTOR protein levels in human Jurkat T cells **A.** Arithmetic means ± SEM (*n* = 6) of miR-15b over 5S rRNA transcript levels in human Jurkat T cells following a 72 hours incubation without (green bar) and with 10 μM (red bar) EGCG. ***(*p* < 0.001), indicates statistically significant difference. **B.** Representative tracings showing the 340/380 nm fluorescence ratio reflecting cytosolic Ca^2+^ activity in Fura-2, AM loaded human Jurkat T cells incubated for 72 hours without (green curve) and with 10 μM (red curve) EGCG followed by subsequent exposure to Ca^2+^-free HEPES, additional exposure to sarcoendoplasmatic Ca^2+^ ATPase (SERCA) inhibitor thapsigargin (Tg, 1 μM) and re-addition of extracellular Ca^2+^ (Ca^2+^ Std HEPES). **C.** Arithmetic means ± SEM (*n* = 5) of the slope (left) and peak (right) of the fluorescence ratio change following re-addition of extracellular Ca^2+^ in human Jurkat T cells incubated for 72 hours without (green bars) and with 10 μM (red bars) EGCG. ***(*p* < 0.001), indicates statistically significant difference. **D.** Original Western blots (left panels) and **E**. arithmetic means ± SEM (*n* = 4-9, right panels) of mTOR/GAPDH and PTEN/GAPDH protein abundance in human Jurkat T cells incubated for 72 hours without (green bars) and with 10 μM (red bars) EGCG. **(*p* < 0.01), ****(*p* < 0.0001) indicates statistically significant difference.

Further experiments were performed to test whether a similar mechanism can affect human T cells. In agreement, to what was shown in murine CD4^+^ T cells, 10 μM EGCG up-regulated the miR-15b in Jurkat T cells (Figure 7A). T cells activation triggers intracellular Ca^2+^ release. Therefore, we quantified SOCE in Jurkat T cells in presence and absence of 10 μM EGCG. A 72 hours treatment of Jurkat T cells with 10 μM EGCG significantly decreased SOCE (Figure 7B & 7C). Moreover, a 72 hours treatment of Jurkat T cells with 10 μM EGCG was followed by statistically significant decrease of mTOR and PTEN protein levels (Figure 7D & 7E).

### EGCG decreases the mitochondrial membrane potential in both human Jurkat T cells and activated murine CD4^+^ T cells

**Figure 8:**
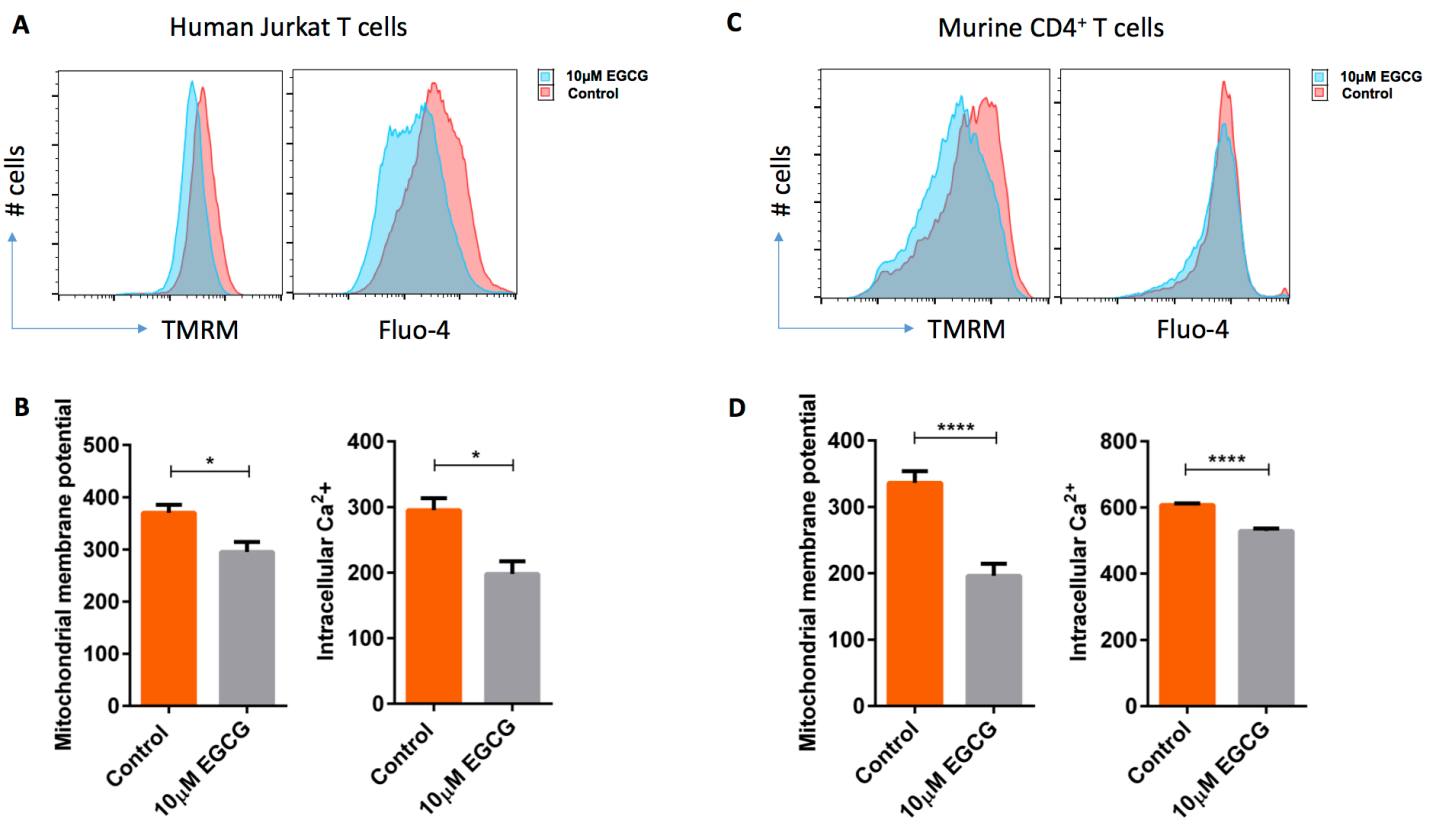
EGCG treatment significantly depolarized mitochondrial membrane and significantly decreased intracellular Ca ^2**+**^ concentration in human Jurkat T cells and murine CD4**^+^** T cells. **A.** Human Jurkat T cells were cultured in the presence of 10 μM EGCG for 3 days. The mitochondrial membrane potential (MMP) and intracellular Ca^2+^ concentrations were measured by flow cytometry. **B.** Arithmetic means ± SEM (*n* = 4-6) of a significant difference in MMP and intracellular Ca^2+^ concentration was observed between control (orange bars) and 10 μM EGCG (grey bars) treated cells. *(*p* < 0.05) indicates statistically significant difference. **C.** Murine CD4^+^ naïve T cells were activated in the presence of anti-CD3/anti-CD28 and cultured in the presence of 10 μM EGCG for 3 days. Mitochondrial membrane potential (MMP) and intracellular Ca^2+^ concentrations were measured by flow cytometry. **D.** Arithmetic means ± SEM (*n* = 6) of MMP and intracellular Ca^2+^ concentration without (orange bars) and with 10 μM (grey bars) EGCG treatment. ****(*p* < 0.0001) indicates statistically significant difference.

Previous observations proposed that EGCG-induced apoptosis is paralleled by mitochondrial depolarization [18, 29]. Therefore, we measured the mitochondrial membrane potential after 72 hours treatment with and without EGCG in Jurkat T cells and murine CD4^+^ T cells. As a result, EGCG treatment significantly decreased mitochondrial membrane potential as well as intracellular Ca^2+^ concentration in human Jurkat T cells (Figure 8A & 8B) and murine CD4^+^ T cells (Figure 8C & 8D).

## DISCUSSION

The present study demonstrates that green tea polyphenol (EGCG) is a negative regulator of store operated Ca^2+^ entry (SOCE) into murine CD4^+^ T cells and human Jurkat T cells [29]. SOCE was lower in the EGCG treated CD4^+^ T cells compared with control CD4^+^ T cells. SOCE is accomplished by Orai1, which is activated by STIM2 [6, 30, 31]. Orai1 expression was lower in EGCG treated CD4^+^ T cells when compared to the control CD4^+^ T cells, an effect paralleled by a similar decrease of SOCE. Therefore, EGCG attenuates Ca^2+^ entry into murine CD4^+^ T cells through down-regulation of both STIM2 and Orai1. An effect of EGCG on SOCE has already been observed earlier [8, 32]. The present observations uncover, however, a completely novel mechanism contributing to or even accounting for the effect of EGCG on SOCE (both murine and human), i.e. the up-regulation of miR-15b, which in turn down-regulates STIM2 and Orai1 at mRNA transcript and protein levels as well as SOCE. Similarly, our unpublished data suggest that overexpression of miR-15b decreases Orai1 protein level and attenuates SOCE in human endometrial adenocarcinoma cells. Hence, EGCG can be considered an epigenetic regulator of the key players accomplishing SOCE in murine CD4^+^ T cells, Jurkat T cells and other cancer cells, i.e. STIM1/2 and Orai1.

Previously, it was reported that EGCG was involved in the induction of apoptosis as well as reduction in cell proliferation in T cells and cancer cells [13, 15, 16, 18, 25, 28]. Our experiments support the previously published findings and suggest that EGCG indeed influences apoptosis and cell proliferation at higher concentrations (20 and 50 μM) [16], which are cytotoxic for normal cells [33]. Previously published studies reported seemingly nontoxic serum concentrations of EGCG of 7.3 ± 3.6 μM in humans and of 20 μM in rats [34] following drinking a few cups of green tea or taking tablets [33]. In this study, experiments were mainly performed at 5 μM - 10 μM EGCG which down-regulated SOCE and up-regulated miR-15b expression.

The miRNAs affect gene regulation at the post-transcriptional level, and thus, changing protein expression stability. Recently, we have shown that miRNAs processing protein Dicer is involved in the regulation of SOCE in murine CD4^+^ T cells [35]. According to previous studies in cancer (MCF-7) cells EGCG up-regulates the expression of miR-16, a member of the miR-15b family (family of miR-16/miR-15a/miR-497/miR-322/miR-195) and consequently, EGCG down-regulates Bcl-2 expression level and thus counteracts cancer progression [25]. Down-regulation of SOCE would similarly compromise cell proliferation [36]. The miR-15b family member, miR-15b/16 is up-regulated in regulatory T cells (Tregs) and required for Tregs development and function [37]. In a similar fashion, EGCG also induces Tregs development [9]. Indeed, miR-15b is also up-regulated in murine CD4^+^ T cells as well as in Jurkat T cells by EGCG treatment. Therefore, miR-15b could be involved in the regulation of Ca^2+^ entry and thus Ca^2+^ sensitive cellular functions such as gene expression, proliferation, cell motility and cytokine expression.

The tumor suppressor activity of PTEN is a lipid phosphatase [38], it dephosphorylating the lipid second messenger phosphatidylinositol 3,4,5- trisphosphate thus antagonizing the phosphoinositide 3-kinase-Akt pathway and preventing activating phosphorylation of Akt [39–44]. A segment of the PTEN protein which are localizes to the endoplasmic reticulum (ER) and mitochondria-associated membranes, signaling domains involved in Ca^2+^ transfer from the ER to mitochondria and apoptosis induction [38]. PTEN silencing impairs ER Ca^2+^ release, lowers cytosolic and mitochondrial Ca^2+^ transients and decreases cellular sensitivity to Ca^2+^-mediated apoptotic stimulation [38]. Alterations in Ca^2+^ signaling in cancer cells promote survival and establish resistance against cell stress and damage, so that the on-going oncogenic stress does not result in the activation of cell death [22, 45]. EGCG dose-dependently induced mitochondrial depolarization, an effect subsequently reversed to a persistent hyperpolarized mitochondrial state dependent on the activity of respiratory Complex I [29]. Furthermore, the effect on MMP depends on the time period of EGCG treatment [29, 46]. In addition, our data further suggest that EGCG decreases PTEN activity similarly in human Jurkat T cells, an effect paralleled by low MMP and low SOCE. Moreover, EGCG treatment also leads to mTOR inhibition, which could contribute to the effect on cell proliferation and apoptosis.

In conclusion, the present observations reveal a novel role of green tea polyphenol EGCG in the regulation of Ca^2+^ entry into murine CD4^+^ T cells and human leukaemic T cell lymphoblasts. EGCG up-regulates the expression of miR-15b which in turn decreases SOCE by down-regulating STIM2 or Orai1 expression. Therefore, our results suggest that up-regulation of miR-15b by EGCG curtails store operated Ca^2+^ entry in murine CD4^+^ T cells and human leukaemic T cell lymphoblasts.

## MATERIALS AND METHODS

### Mice

Cells were isolated from C57BL/6 mice between 8-16 weeks of age (both male and female mice were used for experiments). All experiments were performed according to the EU Animals Scientific Procedures Act and the German law for the welfare of animals. All procedures were approved by the authorities of the state of Baden-Württemberg.

### Murine CD4^+^ T cells isolation and culture and treatment

Murine CD4^+^ naive T cells were isolated from C57BL/6 mice using the MagniSort^®^ Mouse naïve T cell Enrichment kit (#8804-6824-74, eBioscience, Germany) as described by the manufacturer. Purified T cells were cultured in plate-bound anti-CD3 (#16-0031-85, eBioscience)/anti-CD28 (#16-0281-85, eBioscience) Abs at a 1:2 ratio (1μg/ml anti-CD3 and 2μg/ml anti-CD28) in the presence or absence of 5-50 μM EGCG (#E4143, Sigma, Germany) for 3 days.

### Human leukaemic T cell lymphoblasts (Jurkat T cells) culture and treatment

Human Jurkat E6.1 Cell Line (#88042803, Sigma) were maintained in RPMI 1640 (#61870-010, Life Technologies, Germany) medium supplemented with 10% Fetal bovine serum (#10270-106, Life Technologies), 1% penicillin/streptomycin (#P4333, Sigma) and 0.1% 2-Mercaptoethanol (#31350-010, Life Technologies) in cell culture flasks, then incubated at 37°C in a humidified atmosphere at 5% (v/v) CO_2_. Cells were routinely passaged thrice weekly. Jurkat T cells were treated with or without 10 μM EGCG for 3 days. The experiments were performed within 6 months following purchase of the cells.

### Calcium measurement

Fluorescence measurements were performed using an inverted light incidence fluorescence phase-contrast microscope (Axiovert 100, Zeiss, Germany). Cells were excited alternatively at λ = 340 or 380 nm and the light deflected by a dichroic mirror into either the objective (Fluor 40×/1.30 oil, Zeiss) or a camera (Proxitronic, Germany). Emitted fluorescence intensity recorded at λ = 505 nm and data were acquired by using specialized computer software (Metafluor, Universal Imaging, USA) [30].

Cells (either activated CD4^+^ T cells or Jurkat T cells) treated with EGCG for 3 days were loaded with 2 μM Fura-2, AM (#F1221, Molecular Probes, USA) for 30 min at 37°C in a CO_2_ incubator. To measure SOCE, changes in cytosolic Ca^2+^ activity ([Ca^2+^]i) were monitored following depletion of the intracellular Ca^2+^ stores. In brief, [Ca^2+^]i was measured using Ca^2+^ containing standard HEPES buffer [125mM/L NaCl, 5mM/L KCl, 1.2 mM/L MgSO_4_*7H_2_O, 32.2 mM/L HEPES, 2mM/L Na_2_HPO_4_*2H_2_O, 5mM/L Glucose, 1mM/L CaCl_2_*2H_2_O; pH = 7.4] for 2 minutes and then changed to Ca^2+^-free HEPES buffer [125mM/L NaCl, 5mM/L KCl, 1.2 mM/L MgSO_4_*7H_2_O, 32.2 mM/L HEPES, 2mM/L Na_2_HPO_4_*2H_2_O, 5mM/L Glucose, 0.5 mM/L EGTA; pH = 7.4] for 3 minutes. In the absence of Ca^2+^, the intracellular Ca^2+^ stores were depleted by inhibition of the sarcoendoplasmatic Ca^2+^ ATPase (SERCA) by 1 μM Thapsigargin (#67526-95-8, Sigma) and [Ca^2+^]i was measured for another 5 minutes. In the following, Ca^2+^ containing HEPES buffer was added for 5 minutes, which allowed assessing the SOCE.

### Transfection of murine CD4^+^ T cells by miR-15b

Murine CD4^+^ naïve T cells were seeded on a coated 24-well plate. T cells were transfected with miRNA-negative mimic (#479903), cel-miR-39-3p (#479902), miR-15b-mimic (#471275) and mmu-miR-710 (#470077) (all miRNAs mimics, control and inhibitors from Exiqon, Denmark) using DharmaFECT3 (#T-2003-01, Dharmacon, USA) as recommended by manufacture's guidelines. Briefly, Naïve CD4^+^ T cells were prepared in antibiotics free cell buffer and 0.75×10^6^ - 1×10^6^ cells per well cultured in the presence of 500 μl of R-10 medium (RPMI 1640 (#61870-010, Life Technologies) medium supplemented with 10% Fetal bovine serum (#10270-106, Life Technologies), 1% L-Glutamine (#G7513 200mM solution, Sigma), 1% penicillin/streptomycin (#P4333, Sigma) and 0.1% 2-Mercaptoethanol (#31350-010, Life technologies)). Whilst plating the cells, 2 μl of 50 μM stock concentration of non-targeting miRNA-negative mimic, cel-miR-39-3p, miR-15b-mimic and mmu-miR-710 were added to 8 μl of antibiotic free RPMI1640 medium and the miRNAs were incubated for 5 minutes in tube 1. In tube 2, 0.5 μl of DharmaFECT3 was added to 9.5 μl of antibiotic free RPMI1640 medium. The content of tube 1 was added to tube 2 and incubated for additional 20 minutes. After 20 minutes of incubation, the reaction mixture from tube 2 was added to corresponding wells to negative mimic, cel-miR-39-3p, miR-15b-mimic and mmu-miR-710 wells. Cells were further incubated for additional 72 hours then used for qRT-PCR, immunoblotting, and determination of SOCE.

### mRNA and miRNA qRT-PCR

Total RNA including miRNAs was extracted from murine CD4^+^ T cells using miRNAeasy Kit (#217004, Qiagen, Germany). The mRNA (1 μg) and miRNAs (100 ng) were separately reverse transcribed using Superscript III First-Strand synthesis system (#18080-51, Invitrogen, Germany) and miRNA universal cDNA synthesis kit II (#203301, Exiqon) for reverse transcript PCR (RT-PCR) and subsequent real-time quantitative PCR (qRT-PCR). Detection of gene expression was performed with KapaFast-SYBR Green (#KAPBKK4606, Peqlab, Germany) and measurements were performed on a BioRad iCycler iQ^TM^ Real-Time PCR Detection System (Bio-Rad Laboratories, Germany). The relative expression levels of mRNAs were normalized to that of *GAPDH*, whereas the relative expression levels of miRNAs were normalized to that of 5S rRNA. The following primers were used to detect *STIM2 and Orai1* expression. For amplification of different miRNAs, hsa-miR-15b-5p LNA™ PCR primer set (#204243, Exiqon), and reference 5S rRNA primer set (#203906, Exiqon) were used and the reaction was set up as recommended by Exiqon or described earlier [47, 48].

STIM2-F 5’-TGTCTGTGTCAAGTTGCCCT-3’

STIM2-R 5’-TGTCTGGCACTTCCCATTGT-3’

Orai1-F 5’- CCTGGCGCAAGCTCTACTTA-3’

Orai1-R 5’- CATCGCTACCATGGCGAAGC-3’

GAPDH-F 5’-CGTCCCGTAGACAAAATGGT -3’

GAPDH-R 5’-TTGATGGCAACAATCTCCAC-3’

### Immunoblotting

Murine CD4^+^ T cells were activated in presence of anti-CD3 (1μg/ml)/anti-CD28 (2μg/ml) and treated with 10 μM EGCG. After 72 hours of activation and treatment, CD4^+^ T cells were washed once with PBS (#D8537, Sigma) and equal amounts of H_2_O and 2×Lammelli's Buffer for cell lysis were added. Proteins were denatured at 95°C for 5 minutes and stored at -20°C. Sample proteins were loaded on 8% or 10% gel depending on protein size and run at 80-120 V for 90-100 minutes. Proteins were electro-transferred onto PVDF membranes. Membranes were probed with the indicated primary antibodies for STIM2 (1:1000; #4917S, Cell Signaling Technology, Germany), Orai1 (1:1000; #13130-1-AP, Proteintech, United Kingdom) and GAPDH (1:2000; #5174S, Cell Signaling Technology), followed by HRP-conjugated secondary antibodies (1:1000; #7074P2, Cell Signalling Technology). Membranes were washed thrice and the antibodies visualized with enhanced chemiluminescent HRP substrate (#R-03031-D25 and R-03025-D25, advansta, USA).

After 72 hours of treatment with 10 μM EGCG, Jurkat T cells were washed once with PBS and equal amounts of H_2_O and 2×Lammelli's Buffer for cell lysis added. Proteins were denatured at 95°C for 5 minutes and stored at -20°C. Sample proteins were loaded on 8% or 10% gel depending on protein size and run for 80-120 V for 90-100 minutes. Proteins were electro-transferred onto PVDF membranes. Membranes were probed with the indicated primary antibodies for mTOR (1:1000; #2983S, Cell Signaling Technology), PTEN (1:1000; #7960S, Cell Signaling Technology) and GAPDH, followed by HRP-conjugated secondary antibodies. Membranes were washed thrice and antibodies visualized with enhanced chemiluminescent HRP substrate and bands were quantified using ImageJ software (National Institute of Mental Health, USA).

### Apoptosis analysis

Murine CD4^+^ T cells were activated and treated as described in above (Murine CD4^+^ T cells isolation and culture and treatment section). The percentage of apoptotic cells was estimated by flow cytometry using the AnnexinV apoptosis detection kit FITC (#88-8005-72, eBioscience) in accordance with the manufacturer's instructions. Briefly, cells were collected and washed with PBS and 1×binding buffer, respectively, then cells were suspended in 1×binding buffer containing Annexin V-FITC solution (1:50 dilution). After that, cells were incubated at room temperature for 15 minutes, protected from light, and washed with 1×binding buffer again. After adding Propidium Iodide solution (1:100 dilution), cells were incubated at room temperature in the dark for 10 minutes prior to flow cytometry for cell apoptosis analysis. Data were analysed by Flowjo software (FLOWJO, LLC, USA).

### CFSE staining

The proliferation of murine CD4^+^ T cells was detected by CellTrace^TM^ CFSE Cell Proliferation Kit (#C34554, eBioscience). Briefly, cells were washed with PBS once, stained with CellTrace^TM^ CFSE (1:1000 dilution) and re-suspended gently, incubated at 37°C for 15 minutes in the dark, then washed with R-10 medium twice, and CD4^+^ T cells with CFSE dye were activated and treated with EGCG as described in above (Murine CD4^+^ T cell isolation and culture and treatment section). After 72 hours, cells were collected to perform the flow cytometry. Data were analysed by Flowjo software.

### Measurements of intracellular calcium and mitochondrial membrane potential by flow cytometry

After 72 hours of treatment with or without 10 μM EGCG, Jurkat T cells or murine CD4^+^ T cells were washed once and re-suspended in 96 well plates with 200 μL PBS. In the following 1 μM Fluo-4 (#F14200, Invitrogen) or 100 nM Tetramethylrhodamine (TMRM) (# I34361, Invitrogen) were added. Cells were incubated for 30 minutes at 37°C in the dark, then washed with PBS buffer twice. Finally, cells were placed in 200 μL PBS prior to flow cytometry for measurement. Data were analysed by Flowjo software.

### Statistics

Data are provided as means ± SEM, n represents the number of independent experiments. All data were tested for significance using unpaired Student's t-test and ANOVA. Data were analysed by Excel 2013 and GraphPad Prism Software, USA. P value ≤0.05 was considered statistically significant.

## Abbreviations

EGCG: epigallocatechin-3-gallate
SOCE: store operated Ca^2+^ entry
AMPK: adenosine 5’-monophosphate-activated protein kinase
PI3K: phosphoinositide 3-kinase
mTOR: mammalian target of rapamycin
STIM1/2: stromal cell-interaction molecules (STIM) 1 and 2
IP_3_: inositol trisphosphate
CRAC: calcium release-activated calcium
Tg: Thapsigargin
MMP: mitochondrial membrane potential
PTEN: phosphatase and tensin homolog deleted on chromosome 10
ER: endoplasmic reticulum
miRNAs: micro-ribonucleic acids
ROS: reactive oxygen species
PBS: phosphate buffer saline
TMRM: Tetramethylrhodamine.
